# Investigation of Electrically Evoked Auditory Brainstem Responses to Multi-Pulse Stimulation of High Frequency in Cochlear Implant Users

**DOI:** 10.3389/fnins.2020.00615

**Published:** 2020-06-30

**Authors:** Ali Saeedi, Werner Hemmert

**Affiliations:** ^1^Department of Electrical and Computer Engineering, Technical University of Munich, Munich, Germany; ^2^Munich School of Bioengineering, Technical University of Munich, Garching, Germany

**Keywords:** multi-pulse stimulation, temporal integration, brainstem response, cochlear implants, threshold

## Abstract

We investigated the effects of electric multi-pulse stimulation on electrically evoked auditory brainstem responses (eABRs). Multi-pulses with a high burst rate of 10,000 pps were assembled from pulses of 45-μs phase duration. Conditions of 1, 2, 4, 8, and 16 pulses were investigated. Psychophysical thresholds (THRs) and most comfortable levels (MCLs) in multi-pulse conditions were measured. Psychophysical temporal integration functions (slopes of THRs/MCLs as a function of number of pulses) were −1.30 and −0.93 dB/doubling of the number of pulses, which correspond to the doubling of pulse duration. A total of 15 eABR conditions with different numbers of pulses and amplitudes were measured. The morphology of eABRs to multi-pulse stimuli did not differ from those to conventional single pulses. eABR wave eV amplitudes and latencies were analyzed extensively. At a fixed stimulation amplitude, an increasing number of pulses caused increasing wave eV amplitudes up to a certain, subject-dependent number of pulses. Then, amplitudes either saturated or even decreased. This contradicted the conventional amplitude growth functions and also contradicted psychophysical results. We showed that destructive interference could be a possible reason for such a finding, where peaks and troughs of responses to the first pulses were suppressed by those of successive pulses in the train. This study provides data on psychophysical THRs and MCLs and corresponding eABR responses for stimulation with single-pulse and multi-pulse stimuli with increasing duration. Therefore, it provides insights how pulse trains integrate at the level of the brainstem.

## Introduction

Cochlear implants (CI) can restore hearing and speech understanding to people with severe to profound hearing loss to a surprisingly high degree by electrical stimulation of the residual auditory nerves (ANs). As the dynamic range of electric stimulation is much narrower than in the intact ear, it is necessary to set sensation thresholds and maximum stimulation levels properly. Both levels depend on the stimulation rate and on the number of pulses (or the length of the pulse train) delivered. These two parameters which contribute in temporal phenomena are known as multi-pulse integration (MPI) and temporal integration (TI) functions. For a fixed (usually long) stimulation duration, the MPI function is referred to the function relating the psychophysical detection threshold (THR) with stimulation rate (McKay and McDermott, 1998). The TI function describes how the detection THR varies as a function of stimulation duration when the stimulation rate is fixed. The time range in TI functions varies from tens of milliseconds to hundreds of milliseconds with large individual variations. TI in acoustic hearing leads to a THR decrease with a slope of approximately 2.5 dB per doubling of stimulus duration up to about 300 ms (Gerken et al., 1990).

Studies which investigated TI functions for electric hearing generally claimed that, similar to MPI functions, TI slopes drop when the stimulation duration (or equivalently the number of pulses) increased, both in animal studies (Donaldson et al., 1997; Zhou et al., 2015) and in human studies (Zhou et al., 2015). Donaldson et al. (1997) found THR TI slopes of 0.42 dB/doubling of number of pulses, ranging from 1 to 64 pulses at a 100-pps stimulation rate. Zhou et al. (2015) found that for a stimulation rate of 640 pps, mean TI slopes dropped about 0.88 dB/doubling of stimulation duration from 31.25 to 250 ms (20 to 160 pulses). Donaldson et al. (1997) found that not only THRs but also loudness levels including maximum acceptable levels (MAL) dropped when the stimulation duration increased. For MALs, they found large intersubject variabilities of TI slopes, i.e., shallower, equally steep, and steeper TI slopes in comparison to the THR TI slopes. Obando Leitón (2019) measured TI functions for two rates in a very comprehensive study. Slopes showed a large variation between subjects but also for different electrodes within a subject. For a stimulus of 300-ms duration, slopes ranged from −5.24 dB to −2.32 dB/doubling, when stimulation rate increased from 1500 to 18000 pps. Over all subjects, Obando Leitón (2019) observed that increasing the stimulation rate from 1500 to 18000 pps caused THR levels to decrease by approximately 11 dB, which corresponds to a decrease of −3.1 dB/rate doubling. Obando Leitón (2019) also found that the MALs dropped by 4 dB when the stimulation rate was increased from 1500 to 18000 pps, which suggests a slope of −1.11 dB/rate doubling. Temporal integration effects between two pulses are usually quite small (Karg et al., 2013). Nevertheless, for long pulse trains MPI effects on THR and MAL can be large.

For low stimulation rates (below 1000 pps), THRs in CI users fall only by less than 1 dB/doubling of stimulus duration (Donaldson et al., 1997) when the stimulation rate is below 1000 pulses per second (pps). When the stimulation rate exceeds 1000 pps, the slope of the MPI function becomes steeper, in guinea pigs (Middlebrooks, 2004; Kang et al., 2010; Zhou et al., 2015) and in humans (Shannon, 1985; McKay and McDermott, 1998; Zhou et al., 2012; Carlyon et al., 2015). As an example, Kang et al. (2010) found a significant decrease in MPI slopes when rates below 1000 pps increased to above 1000 pps at two stimulation sites (Δslopes = −2.88 and −2.83 dB/doubling of pulse rate at two stimulation sites). Similarly, Carlyon et al. (2015) observed a THR decrease of 7.71 dB when increasing the stimulation rate from 500 to 3500 pps for pulse durations of 400 ms, which is equivalent to a slope of −2.74 dB/rate doubling. An exception was Skinner et al. (2000), who found the MPI slope to drop by less than 0.1 dB/doubling of the pulse rate for rates above 1000 pps and even less for rates below 1000 pps. Slopes of MPI functions for C-levels are reported to be steeper for rates above 1000 pps compared to rates below 1000 pps (Zhou et al., 2012). In a human study, they found that MPI slopes for the C-levels were 0.65 dB, 0.54 dB, and 1.19 dB/doubling or the stimulation rate is steeper for rates above 1000 pps compared to rates below 1000 pps, respectively, for three stimulation sites. Zhou et al. (2012) observed that TI slopes for THRs were steeper than those for MAL/C levels. For basal and middle sites, MPI slopes for THRs were 1.24 dB and 1.07/doubling of the rate, respectively, which were 0.59 dB and 0.53 dB steeper than their corresponding MPI slopes for C-levels. Since Zhou et al. (2012) found no correlation between slopes of C-level and THR MPI functions, they claimed that the underlying mechanisms of these two functions are probably different.

Middlebrooks (2004) and Zhou et al. (2012) attributed the steeper MPI slopes at rates above 1000 pps to a residual partial depolarization mechanism, where initial subthreshold pulses partially depolarize a single AN or a group of ANs and further pulses, accruing within a 1-ms time window, increase the chance of firing an action potential, thus lowering the THR level. In terms of temporal considerations, this effect is also known as “facilitation,” where the elevated membrane potential of the auditory nerve, as the effect of the first pulse in the train, facilitates it for the successive pulses to elicit an action potential (Hodgkin, 1938; Hodgkin and Huxley, 1952; Boulet et al., 2016).

The slopes of MPI functions are suggested to be possibly an indicator of cochlear health in the area close to the stimulation site, either in CI users (Kang et al., 2010; Pfingst et al., 2011; Zhou et al., 2012, 2018; Zhou and Pfingst, 2016) or in normal-hearing listeners (Shannon, 1983). Psychophysical results from Kang et al. (2010); Pfingst et al. (2011) indicated that in guinea pigs, for stimulation rates below 1000 pps, there is a correlation between the THR MPI slopes and cochlear health state in terms of hair cell counts, auditory nerves, and ensemble spontaneous activity (ESA).

Electrical stimulation with high pulse rates are thought to resemble the spontaneous activity of ANFs in a healthy ear (Rubinstein et al., 1999; Litvak et al., 2003; Hughes et al., 2012). Rubinstein et al. (1999) found that for pulse rates above 2000 pps, human electrically evoked auditory compound action potential (eCAP) responses to a pulse train dramatically dropped after a strong response to the initial pulse of the train and sustained afterward. They interpreted this sustained activity as an independent quasi-stochastic activity of ANFs resulting from desynchronization of populations of ANFs. For stimulation rates below 1016 pps, they still observed an alternating amplitude pattern of the eCAP for successive pulses of the train after a relatively strong initial response to the first pulse. The rate at which the alternating pattern seemed to vanish and the sustained pattern appeared was referred to as “stochastic rate” (Hughes et al., 2012) and occurred at rates above 2033 pps in Rubinstein et al. (1999). Hughes et al. (2012) observed that the stochastic rate was variable (about 2400 to 3500 pps) between different electrodes in human subjects. Similar to human results, Litvak et al. (2003) found a sustained discharge rate in cat ANFs in response to a 5000-pps pulse train. They claimed that, since no correlation between simultaneous measurements of pairs of ANF activities was found, the 5000-pps pulse rate desynchronized the auditory nerve activities, which is, again, evidence that high stimulation rates could improve neural representation to electric stimuli.

Another motivation to use high pulse rates in electric hearing is to represent the global stimulation rate induced by the stimulation rate of individual electrodes in CIs. Results of the finite element model from Bai et al. (2019) and measurement data from Obando Leitón (2019) and many others suggest that stimulation of a single electrode contact leads to a broad spread of current along the cochlea, which means that in electric hearing, neurons are stimulated not only by the nearest electrode but also by the neighboring electrodes. Therefore, the effective stimulation which reaches a spiral ganglion neuron—at least in the continuous interleaved stimulation (CIS) strategy—is a burst with the global stimulation rate originating from neighboring electrodes, which is very similar to our experiment.

The studies mentioned above investigated the effects of multi-pulse stimulation on either most central (psychophysical studies) or most peripheral (eCAPs or ESA) stages of the auditory system. It is still worth investigating such an effect at a location between these two extreme regions, which, to our best knowledge, has not yet been done. Such a study will shed light on the temporal integration at the level of the auditory brainstem as well as on how temporal properties such as refractoriness and facilitation would function. Based on these foundations, we designed this study to investigate electrically evoked auditory brainstem responses (eABRs) to high rate electrical multi-pulse stimuli in CI users. We measured eABRs to the stimuli with different number of pulses but with the same physical stimulation amplitude to see how multi-pulses are integrated in the level of the brainstem. We also evaluated the contribution of nerve responses to each pulse or to a few consecutive pulses in multi-pulse stimulation to estimate the post-stimulus time histogram (PSTH) of the nerve.

## Materials and Methods

Sixteen ears from twelve participants (two males, mean age: 56.5 years) implanted with Med-El CIs were measured (Table 1). Amplitude growth functions in MP conditions were measured from 8 ears (out of 16; last column of Table 1). Participants signed a written informed consent form and were paid for their participation. The experiment was approved by the Ethics Committee of Klinikum rechts der Isar, Munich.

**TABLE 1:** Demographic information of CI subjects that participated in the study.

Subject	Side	Age range (years)	Etiology	Dur. deaf (years)	CI use (years)	CI type	Electrode	Data in Figure 11
S1	L	50–55	Inherited OM	49	4	Co	6	Yes
S2	L, R	56–60	Congenital	56	12,10	P,So	6,4	Yes
S3	L, R	60–65	Unknown	22	4.5,5	So,So	4,6	No
S4	L, R	56–60	Unknown	56	11,10	P, P	6,7	Yes
S5	L, R	66–70	Unknown	27	12,6	P, P	7,7	No
S6	L, R	60–65	Meningitis, unknown	32	2,8	Sy, Co	6,5	No
S7	L	56–60	Unknown	44	3	So	6	No
S8	L	40–45	Congenital	42	5	Co	4	Yes
S10	L	76–80	Unknown	30	20	Sy	4	No
S13	L	40–45	OM	40	3	Sy	7	Yes
S14	R	36–40	Inherited OM	31	6	Co	4	Yes

*OM, otitis media; Co, concerto; P, pulsar; So, sonata; Sy, synchrony.*

### Stimuli

In this study, we mainly focused on the analysis of eABR wave eV, which usually occurs at around 4 ms after the stimulus onset. This constrains the stimulation duration to be less than 4 ms; otherwise, stimulus and response would interfere. A further limitation comes from the large stimulation artifact, which follows the stimulus and limits the stimulation window to be even shorter. Therefore, in order to obtain clear eABR peak eVs, we employed a stimulation window of up to 1.6 ms, within which pulse trains of up to 16 pulses with a pulse rate of 10,000 pulses per second (pps) were closely packed together to form multi-pulse stimuli.

**FIGURE 1:**
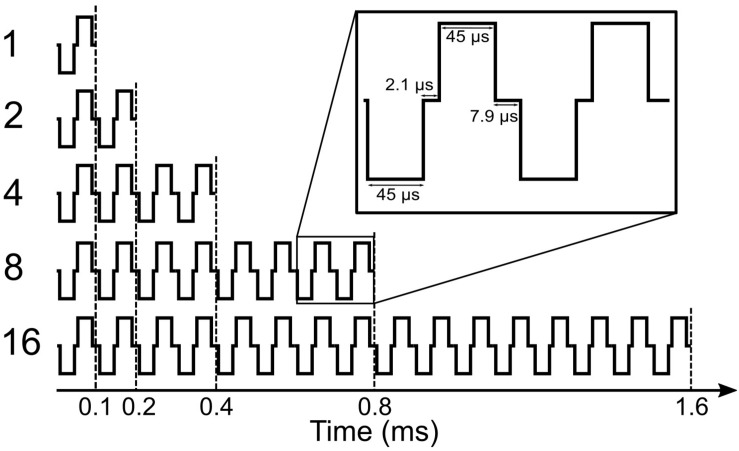
Shape of multi-pulse stimuli used in the study.

An overview of the stimuli is illustrated in Figure 1. Electric pulse trains of 1 pulse, 2 pulses, 4 pulses, 8 pulses, and 16 pulses were used. Pulses were anodic-leading charge-balanced biphasic pulses with a 45-μs phase width and a 2.1-μs interphase gap. Multi-pulse (MP) stimuli were assembled by putting single pulses together with an inter-pulse gap of 7.9 μs to achieve a pulse period of 100 μs and, consequently, a burst rate of 10,000 pps, which is well above standard clinical rates. All MP stimuli were delivered at a repetition rate of 37 Hz through an electrode in the middle of the array (subject specific electrode).

### Pretest

In order to select the stimulation electrode for the experiment, trial psychophysical and eABR measurements were performed on electrode numbers 4, 5, 6, 7, and 8 (out of 12 electrodes in an apical-to-basal order). Psychophysical THRs and MCLs were determined by CI users. The stimulus was single-pulse (1 pulse condition) with the same parameters mentioned above. For each electrode in eABR measurements, the stimulation amplitude was set to 95% of the corresponding psychophysical dynamic range (DR, defined as MCL—THR). The electrode corresponding to the eABR with the largest wave eV amplitude was selected and used for the entire measurements. In case of electrodes with similar eV amplitudes, the one with larger DR was selected.

Once an electrode was determined, psychophysical thresholds (THR) and most comfortable levels (MCL) in MP conditions were adjusted by the subjects while they were seated on a comfortable coach. On a normal keyboard, the subjects used two keys (PgUp and PgDn) for coarse changes and two other keys (up arrow and down arrow) for fine changes. The procedure of adjustment was monitored by the examiners using a custom-designed graphical user interface. In order to avoid any visual biases, subjects did not see the monitor screen. The THRs and MCLs for each MP condition were measured in one trial round and two main rounds. Stimuli were presented randomly, but THR and MCL were measured in separate sessions. For THRs, CI users were asked to raise the stimulation amplitude until they could clearly perceive it and then reduced it so that they could not perceive it any more. For MCL measurements, they were asked to increase the stimulation amplitude to the highest level, which they could still comfortably stand for 3 min. This duration is about three times the duration of a single eABR recording trial. Only the results of the main rounds were used for psychophysical analysis and, later, for eABR measurements. The stimuli used in psychophysical measurements were the same as those employed in eABR measurements.

### eABR Multi-Pulse Stimuli

We call the measured DRs in 1-, 2-, 4-, 8-, and 16-pulse conditions as DR1, DR2, DR4, DR8, and DR16, respectively. Maximum stimulation amplitudes (MSA) were always limited at 95% of the corresponding DRs to avoid very loud stimulation. They were called MSA1, MSA2, MSA4, MSA8, and MSA16, e.g., MSA4 means a stimulation amplitude of 95% of DR4. An exception was subject S14R, where due to a strong artifact at 95% of DRs, 60% was used for all numbers of pulses.

**FIGURE 2:**
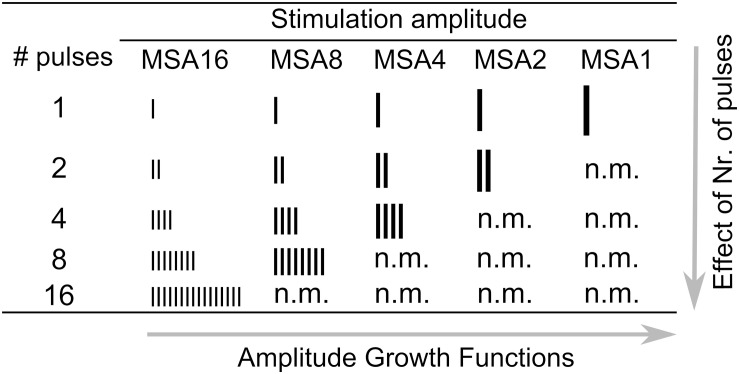
Setup for electrical stimulation via CI and eABR recording.

Figure 2 shows a schematic view of all stimulation conditions used in this study. Different numbers of vertical bars depict the number of pulses, and different bar sizes indicate stimulation amplitudes. Some conditions were not measured (n.m. in Figure 2) because they were above comfortable loudness. In each row of Figure 2, the number of pulses is constant, while the stimulation amplitude varies. Thus, a row-wise investigation of the table provides amplitude growth functions (AFG) of MP conditions. On the other hand, in each column of the table, the stimulation amplitude is constant, while the number of pulses varies. Thus, an investigation of the effect of number of pulses is feasible by column-wise investigation of the table. We also provide eABR amplitude growth functions (AGFs) in MP conditions from 8 ears (out of 16 ears). Stimuli with amplitudes of 5 to 95% corresponding DRs with steps of 10% were used.

### eABR Recording

Stimulation scripts were written in MATLAB and executed on a personal computer equipped with a National Instrument (NI) I/O card. Subjects were asked to remove their speech processors before the measurements, and stimuli were then generated and delivered to CIs via an external induction coil of a research interface box (RIB II), provided by the University of Innsbruck, Innsbruck, Austria.

**FIGURE 3:**
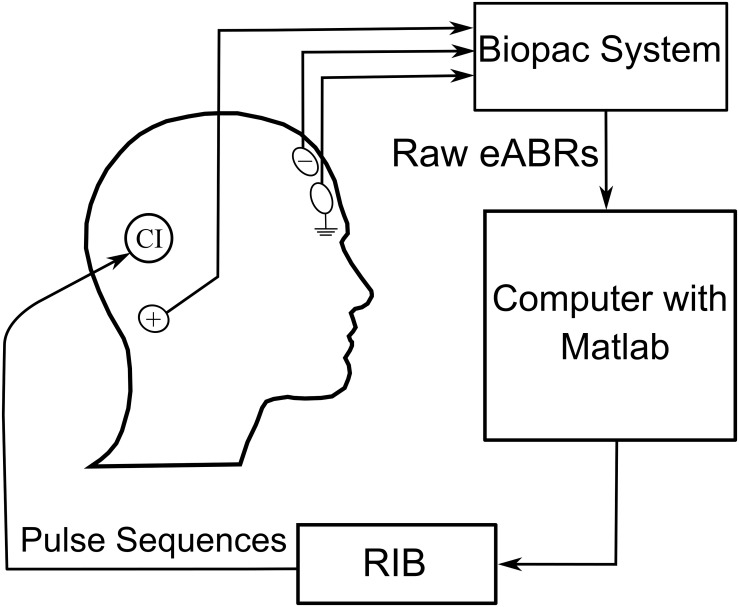
eABR multi-pulse measurement conditions (**n.m.** means not measured).

The stimulation/recording setup is shown in Figure 3. The eABRs were recorded from surface electrodes glued on the skin. The positive electrode was placed behind the ear. The negative and ground electrodes were placed on the upper and lower forehead, respectively. Raw eABRs were recorded with a Biopac^®^ MP36 system (Goleta, CA, United States) with a sampling rate of 100 kHz, a 24-bit A/D converter, and an amplifier gain of 1000. An internally implemented hardware band-pass filter with cutoff frequencies of 0.05 Hz and 20 kHz was used in eABR measurements. No trigger signal was recorded, as the electric stimulation artifact was large enough for stimulus onset detection. For each MP condition, 2184 epochs were recorded, each of which had a duration of 27 ms.

The skin beneath electrodes was cleaned with alcohol swabs, smoothly but thoroughly scrubbed to achieve low-electrode impedances. Conductive gel was used to increase the impedance match between the electrodes and the skin surfaces. Electrode impedances were controlled by the recording setup and were kept below 10 kΩ. During eABR recording, subjects were either sitting or lying on a couch. They were asked to stay as calm as possible to avoid myogenic artifacts. Breaks were taken on regular intervals or on subjects’ demands.

### eABR Processing

Raw eABRs were processed offline using MATLAB R2017b in a series of steps. First, stimulus onset detection was performed using the electrical stimulation artifacts (which were larger than about 300 μV). They were orders of magnitudes higher than neuronal responses (maximum of about 2.6 μV). Using onset indices, data were divided into epochs of 27 ms long. Since most of the eABR information is within the first 10 ms, epoch lengths were reduced to 10 ms. Epochs contaminated with myogenic activities (e.g., eye blink, facial muscle movement) were removed, and only “clean” epochs were used in further analysis. In order to determine the clean epochs, the distribution of the RMS values of epochs was used. For all users, the RMS value of epochs had lognormal distribution. A normal distribution was fitted to the logarithm of the RMS (logRMS) value of epochs. Epochs with logRMS values in the range of μ ± kσ were considered as clean epochs. μ and σ were the mean and standard deviation of the fitted distribution, respectively. The k parameter was subject-specific and varied from 0.7 to 2. Across all subjects, at least 2053 epochs (out of 2184 epochs) remained for averaging.

**FIGURE 4:**
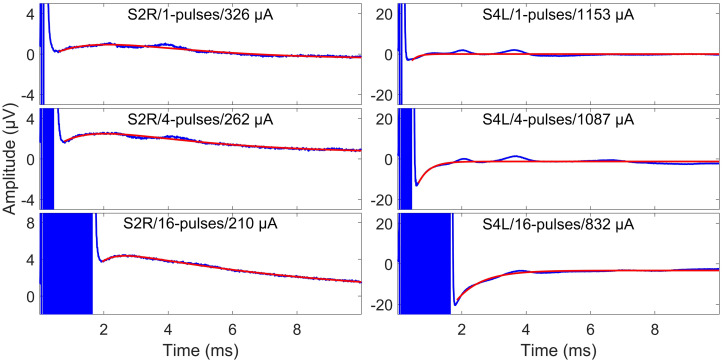
Surface electrode recordings (blue curves) and exponential fittings of stimulation artifacts (only after stimulation, red curves). The left column shows two-exponential fittings, and the right panels show one-exponential fittings. In each panel, the number of pulses and the stimulation amplitude are indicated. Note that the stimulation artifact exceeds the range displayed in the figure.

The next step dealt with electrical artifact suppression. The pattern of the electrical artifacts was subject-dependent. For some subjects, one-exponential fittings worked, while for other subjects, two-exponential fittings were required [blue curves in Figure 4, compared with Spitzer et al. (2006)]. Therefore, exponential functions with the general forms of Eq. (1) and Eq. (2) were used to eliminate electrical artifacts. For each subject, only one function was used for curve fitting, but for each measurement condition, the fitting was performed independently. The decision of using one exponential or two exponentials was made by visual inspection of the discharge curve shape. The starting point of the fitting window varied since the duration of electrical artifacts varied due to different numbers of pulses. Therefore, this parameter was excluded from the fitting curve, as in Hu et al. (2015). The end point of the fitting window was always set to 10.0 ms after the stimulus onset. The fitted artifact was subtracted from the individual eABR epochs.

(1) $$f\,\left(t\right)=a_{0}+a_{1}\,e^{-b_{1}\,t}+a_{2}\,e^{-b_{2}\,t}$$

(2) $$f\,\left(t\right)=a_{0}+a_{1}\,e^{-b_{1}\,t}$$

Noise was reduced by zero-phase digital filtering (band-pass 4th order Butterworth filter, passband: 100 Hz to 3 kHz). As a final stage, weighted non-stationary fixed multi-point (WNSFMP) averaging was applied (Silva, 2009). In this method, the variation of multiple fixed time points in subsets of epochs is analyzed to estimate the variance of the residual noise (RN). The WNSFMP method assumes stationary noise within a subset of epochs, but still lets the noise vary within different subsets. This enables the method to eliminate the effect of non-stationary noise and, on the other hand, to make a weighted averaging with weights being the inverse of corresponding subset variances. The WNSFMP method also provides post-average RN estimation; its variance (${\hat{\sigma }}_{R\,N}^{2}$) is a measure of RN power. In this study, amplitude variances were estimated as ${\hat{\sigma }}_{a\,m\,p}^{2}=2\,{\hat{\sigma }}_{R\,N}^{2}$, as in Undurraga et al. (2013).

**FIGURE 5:**
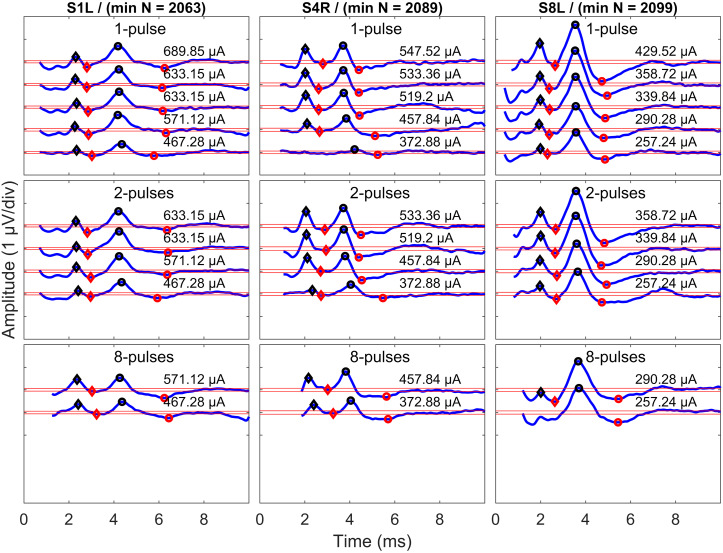
Exemplary final eABRs for three subjects (columns) in multi-pulse conditions (rows). The stimulation amplitudes and the number of pulses are indicated in each panel. Significant peaks and troughs of eIII are marked with filled black and red diamonds, respectively. Peaks and troughs of eV are shown with filled black and red circles, respectively. Horizontal red lines indicate ± $\sqrt{2}\,{\hat{\sigma }}_{R\,N}$. The minimum number of epochs used for averaging (min N) is indicated for each subject.

Only eABR wave eV amplitudes and latencies were analyzed, as wave eIII was corrupted by the stimulation artifact, especially in the 8- and 16-pulse conditions. Wave eV amplitude was calculated as the difference of peak eV and the next trough, and the latency of wave eV was defined as the time point where peak eV occurred. Only amplitudes greater than $\sqrt{2}\,{\hat{\sigma }}_{R\,N}$ were accepted as valid amplitudes and were used for further analysis. Exemplary final eABRs in 1-, 4-, and 8-pulse conditions are shown in Figure 5 for three subjects.

### Statistical Analysis

Repeated-measures analysis of variance (ANOVA) was used to statistically test the effect of the number of pulses. Statistical analysis was performed in MATLAB 2017b. In psychophysical data, the within-subject variable was changed in THRs and MCLs, while in eABR data, the within-subject variable was changes in wave eV amplitudes. For pairwise comparisons, Bonferroni corrected *post hoc* analysis was applied. The statistical significance level was set to α = 0.05.

## Results

### Psychophysical Results

**FIGURE 6:**
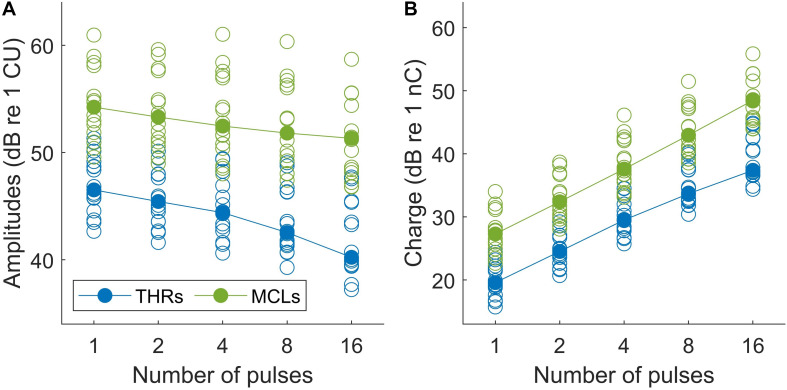
**(A)** Psychophysical THR and MCL currents (in dB re 1 μA) and **(B)** total burst charge (TBC) (dB re 1 nC) for 14 subjects (19 ears). Values differed significantly between all conditions (*p* < 0.05).

Results of psychophysical experiments are plotted in Figure 6. THRs and MCLs are plotted for individual subjects in Figure 6A with open blue and green circles, respectively. Total burst charges (TBCs) used to reach THRs and MCLs are also depicted in filled circles in Figure 6B. The TBC was defined as overall charges in positive phases of multi-pulses. The corresponding median values of each set of the data are shown with filled symbols.

The median THRs and MCLs for single pulses were 211.8 μA and 514.5 μA, respectively, which corresponds to TBCs (of the integrated positive pulse phases) of 9.4 and 23.1 nC, respectively. This corresponds to a dynamic range from 4.65 to 12.61 dB (median: 7.17 dB). With increasing number of pulses, both THRs levels and MCLs decreased monotonically, almost for every measurement and patient, with steeper drops for THRs. The median THR levels over all subjects dropped by about 6.30 dB when the number of pulses increased from 1 to 16 pulses, whereas the decrease for MCLs was only 2.90 dB. For the analysis, linear regression was calculated for each set of data and averaged. The THRs decreased with an average slope of 1.30/doubling of the number of pulses (ranged from 0.65 to 2.34 dB/doubling), while the MCLs decreased with an average slope of 0.93 dB/doubling of the number of pulses (ranged from 0.66 to 1.32 dB/doubling).

Two-way repeated measures of ANOVA showed that THR and MCL data (amplitudes and TBCs) in Figure 6 dropped significantly as a function of number of pulses. In panel A, both THR and MCL decreased significantly [main effect of the number of pulse; *F*(4,112) = 176.14, *p* < 0.001] when the number of pulses increased from 1 to 16. The interaction effects between THRs vs. MCLs were significant [*F*(4,112) = 5.26, *p* < 0.001], which indicates a shallower slope for MCLs compared to THRs. In panel B, THR and MCL TBCs increased significantly [main effect of the number of pulse; *F*(4,112) = 3470.2, *p* < 0.001] as a function of number of pulses. The interaction effects between THRs vs. MCLs were significant [*F*(4,112) = 5.26, *p* < 0.001], which indicates a shallower slope for THR TBCs compared to MCL TBCs.

### eABR Results

**FIGURE 7:**
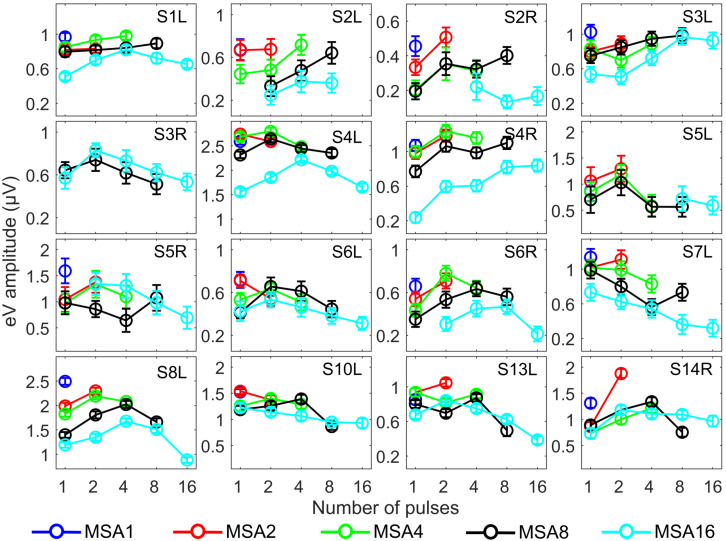
eABR wave eV amplitudes corresponding to the 15 measurement conditions mentioned in Figure 2. Curves with specific colors represent responses to stimuli with fixed stimulation amplitude and varying numbers of pulses. Error bars indicate ± $\sqrt{2}\,{\hat{\sigma }}_{R\,N}$.

**FIGURE 8:**
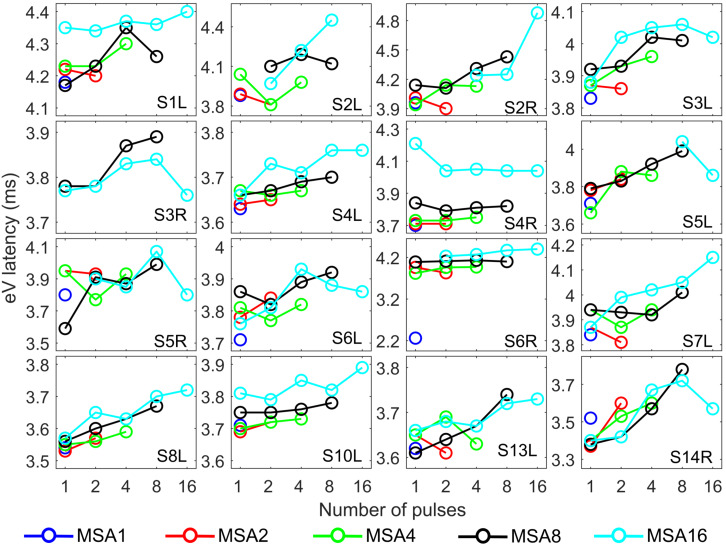
eABR wave eV latencies corresponding to the 15 measurement conditions mentioned in Figure 2. Curves with specific colors represent responses to stimuli with fixed stimulation amplitude and varying numbers of pulses.

**FIGURE 9:**
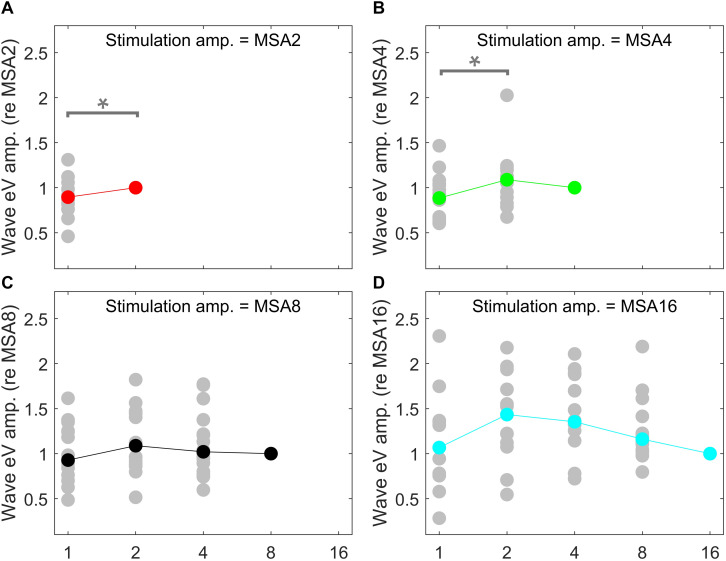
eABR eV amplitudes of multi-pulse conditions over all subjects. In each panel, the stimulation amplitude is constant [MSA2, MSA4, MSA8, MSA16 in panels **(A–D)**, respectively]. Data from individual subjects are plotted in gray circles and their corresponding median values in colors, which match the colors in Figure 7. In each panel, data were normalized to (divided by) the corresponding responses at the largest number of pulses [2, 4, 8, and 16 pulses in panels **(A–D)**, respectively]. Data of the MSA1 condition (blue points in Figure 7) are not plotted, as all were 1 due to normalization. The asterisk shows pairs with significant difference.

Since eABR wave eIII was corrupted by the multi-pulse stimulation artifact especially in measuring conditions with larger number of pulses, we focused on wave eV amplitudes and latencies. Figures 7, 8 show individual eABR wave eV amplitudes and latencies for all CI subjects, respectively. Each panel consists of 15 data points (measurement conditions listed in Figure 2). In each panel, data points with the same color represent responses to stimuli with equal current amplitudes, but with different numbers of pulses. Amplitude growth functions in Figure 7 (reading data for identical numbers of pulses) indicate that eV amplitudes grow generally monotonous with stimulus level. Lines in a single color show how wave eV parameters depend on the number of pulses. Note that because of maximum stimulation levels mentioned earlier, measurement conditions differ in number of data points. Since wave eV amplitude was calculated by subtraction of two values (peak eV and the following trough), error bars in Figure 7 are equal to $\sqrt{2}\,{\hat{\sigma }}_{R\,N}$. No efforts were made to estimate error bars for latencies (Figure 8). Results of eABR eV amplitudes in multi-pulse conditions over all subjects are plotted in Figure 9. In each panel, data were normalized to (divided by) the corresponding responses at the largest number of pulses (2, 4, 8, and 16 pulses in panels A–D, respectively). Data points in gray show individual CI responses to multi-pulses, and the colored circles, which match the colors in Figure 7, are their corresponding median values. Data for MSA1 are not plotted, as all values were 1 due to normalization.

The stimulation amplitudes in MP conditions were 95% of the corresponding DRs for the longest burst. For shorter bursts, however, this stimulation amplitude was far below this value. Over all subjects, stimulation amplitudes of MSA16 (95% of DRs in 16-pulse conditions) corresponded to averages of 35, 46, 60, and 74% of the DRs in 1-, 2-, 4-, and 8-pulse conditions, respectively. Similarly, stimulation amplitudes of MSA8 (95% of DRs in 8-pulse conditions) corresponded to averages of 52, 63, and 78% of the DRs in 1-, 2-, and 4-pulse conditions, respectively. For example, for the 1-pulse conditions, the stimulation amplitudes were at 35, 52, 65, 80, and 95% of the DR (averaged over all subjects; more details are available in Supplementary Figures S1, S2). Visual inspection of the curves from individual CI subjects in Figure 7 shows that intersubject variability is high. Yet, some trends could be detected. For most subjects, and particularly in 8-pulse and 16-pulse conditions, eABR wave eV amplitudes tend to increase when the number of pulses increased from 1 pulse up to a certain number of pulses, i.e., up to 2, 4, or 8 pulses, then they seem to saturate or even decrease. Such an increase was not found for the stimulation amplitude MSA16 (cyan data points in Figure 7) for S7L and S10L, where a monotonically decreasing trend was observed. The points where wave eV amplitudes reached their maximum depended on the subject but also on level within a subject. Due to a facial nerve artifact, eABRs in some conditions were not reliably measured and thus excluded from the dataset (e.g., subject S3R). Similar to the amplitudes, latencies across subjects showed high variability, as depicted in Figure 8. However, for a fixed stimulation amplitude (lines with single colors), the general trend was that latency was increasing with the number of pulses. Moreover, for a fixed number of pulses, higher stimulation amplitudes resulted in shorter latencies, as expected.

Amplitude averaged over all subjects, depicted in Figure 9, suggests that wave eV grows when the number of pulses increased from 1 to 2 pulses and then tended to decrease for further pulses. Statistical analysis on overall results showed a significant difference only between 1- and 2-pulse conditions when the stimulation amplitude was MSA2 [*F*(1,14) = 4.73, *p* < 0.05] (red data points in Figure 9) and MSA4 [*F*(2,28) = 3.66, *p* < 0.02] (green data points in Figure 9).

**FIGURE 10:**
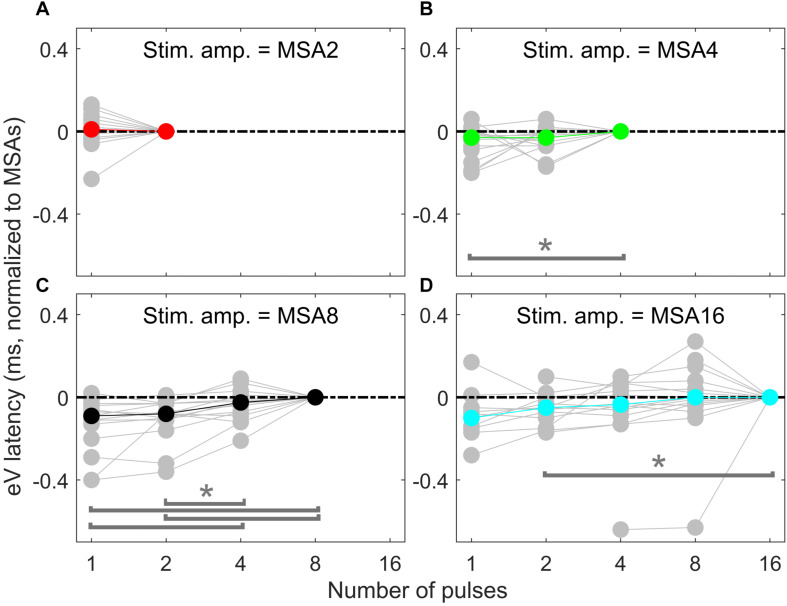
eABR eV latencies of multi-pulse conditions over all subjects. In each panel, the stimulation amplitude is constant [MSA2, MSA4, MSA8, MSA16 in panels **(A–D)**, respectively]. Data from individual subjects are plotted in gray circles and their corresponding median values in colors, which match the colors in Figure 7. Data of the MSA1 condition (blue points in Figure 7) are not plotted, as all were 0 due to normalization. The asterisk shows pairs with significant difference.

Overall results of wave eV latencies corresponding to data in Figure 9 are depicted in Figure 10. Data in each panel were normalized to (subtracted from) the corresponding latencies at conditions with the largest number of pulses, i.e., MSA2, MSA4, MSA8, and MSA16 in panels A to D, respectively. Note that data for MSA1 are not plotted. Statistical analysis shows significant differences between 1 pulse and 4 pulses [*F*(2,28) = 3.15, *p* < 0.05] when the stimulation amplitude was MSA4 and also between four pairs when the stimulation amplitude is MSA8 [*F*(3,42) = 12.29; *p* < 0.01 for 1 pulse and 4 pulses, *p* < 0.01 for 1 pulse and 8 pulses; *p* < 0.02 for 2 pulses and 4 pulses; *p* < 0.01 for 2 pulses and 8 pulses]. In the 16-pulse condition, only the difference between 2-pulse and 16-pulse conditions was significant [*F*(4,40) = 4.80; *p* < 0.05].

**FIGURE 11:**
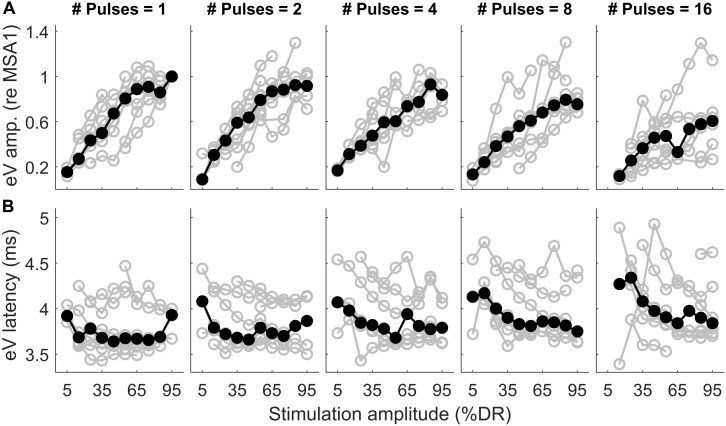
Wave eV amplitude growth functions **(A)** and latency functions **(B)** as a function of stimulation amplitude in all MP conditions for 8 ears (last column of Table 1). The amplitude data was normalized to the largest valid wave eV amplitudes in the 1-pulse condition for each ear. Results from individual subjects are plotted in open gray circles, while the corresponding median values are plotted in filled black circles.

Figure 11 shows wave eV amplitudes and latencies as a function of stimulation amplitudes (%DR) in different MP conditions for 8 ears (out of 16 ears). Columns show results for different numbers of pulses, while top and bottom rows show results of wave eV amplitudes and latencies, respectively. The amplitude data in top panels was normalized to the largest wave eV amplitudes that could be measured in the 1-pulse condition (mostly 95% DR). Data from individual ears are in gray, and the corresponding median values are depicted in black. The median AGFs showed a monotonic increasing trend except for a few cases. Due to the small latency variabilities between subjects, latency data in bottom panels were not normalized. Visual inspection in top panels shows a saturating tendency for the AGFs in MP conditions. The variation of range of eV amplitudes as a function of number of pulses was insignificant only between 2 pulses and 16 pulses [*F*(4,24) = 7.55, *p* < 0.02]. The variation of ranges of eV latencies as a function of number of pulses was significant only between 1 pulse and 8 pulses [*F*(4,24) = 5.24, *p* < 0.02] and between 2 pulses and 8 pulses [*F*(4,24) = 5.24, *p* < 0.03].

The structure of data on AGFs in MP conditions is different from that presented in Figures 9, 10. In the latter, we used fixed stimulation amplitudes for different numbers of pulses, while in the former, the stimulation amplitudes of the same percentage of the DRs were not identical. For instance, the physical stimulation amplitudes at 65% DR in 1, 2, 4, 8, and 16 pulses were not the same. Therefore, we could not apply the same analysis to both datasets.

## Discussion

### Artifact Suppression

In neurophysiological measurements such as eABRs or eCAPs, electrical stimulation artifacts are inevitable. Factors such as stimulation mode, amplitude, phase width, polarity of the stimulus, and stimulation site affect the magnitude and morphology of the stimulation artifact. Low stimulation amplitudes generate small artifacts, it may still be possible to extract eABRs without further processing (Gordon et al., 2008). Often even large artifacts decay rapidly, such that they do not interfere with the eABR waves and blanking of the artifact-contaminated region is sufficient (Tykocinski et al., 1995; Truy et al., 1998). When long and strong artifacts corrupt the eABRs, stimulation with alternating polarity is a further option to reduce artifacts (Abbas and Brown, 1991; Spitzer et al., 2006; Bahmer et al., 2008). However, due to non-linearities of the eABR generation (probably mostly due to the stimulation electrodes), residual artifacts may remain even with alternating polarity stimulation. A different approach was proposed by Bahmer et al. (2010), who measured eABRs in response to triphasic pulses. They varied the distribution of charge over the three phases and selected a configuration, where the artifact was minimal. However, adopting this procedure for pulse train stimulation is not straightforward. In this case as well as when only single polarity stimuli are used, exponential fitting can be used to subtract artifacts (Undurraga et al., 2013; Hu et al., 2015). For stimuli consisting of multi-pulses, accumulated charges remaining from individual pulses yield to higher artifacts compared to single-pulse stimulation. This could be the reason why in this study it became apparent that the stimulation artifacts obviously had two components, which can be fitted by two exponential functions. This was already found in a few studies even for conventional biphasic (Spitzer et al., 2006) or triphasic stimuli (Bahmer and Baumann, 2012). Two-exponential fitting functions used in this study appeared to robustly and reliably remove the artifact even for long stimuli, e.g., 16 pulses, where the artifact superimposed with the eABR wave eV.

### TI Functions in Psychophysical Data

The first part of this study examined the TI functions of THRs and MCLs as a function of stimulation duration, which increased from a single pulse to 1600 μs (16 pulses). As the psychophysical THRs and MCLs in this study were determined for the purpose of eABR measurement, the stimulation pattern differed fundamentally from those usually used for psychophysical measurements in other studies (e.g., McKay and McDermott, 1999; Zhou et al., 2015). In this study, besides the high stimulation rate of 10,000 pps, a repetition (burst) rate of 37 bursts per second was presented, which was essential to record eABRs which require fast averaging. This way, it was possible to apply identical stimuli for both psychophysical measurements and eABR recordings. Nevertheless, even with these deviations in stimulation pattern, results were in line with previous studies. We observed a decrease of −1.31 dB/doubling of stimulation duration in TI slopes of THR levels. If this is combined with the TI slopes of −0.42 dB (Donaldson et al., 1997), −0.88 dB (Zhou et al., 2015), −1.0 dB, and −2.6 dB/doubling the number of pulses (Obando Leitón, 2019), one can see that the TI slopes decrease monotonically when the stimulation rate increased. We also compared the TI slopes of THR levels with those of wave eV amplitudes, for conditions of a fixed-stimulation amplitude (MSA8 and MSA16), while the number of pulses changed, as well as for conditions of a fixed number of pulses, while the stimulation amplitude changed (AGFs in 1-pulse and 2-pulses conditions). Details of these comparisons are available in Supplementary Figures S2–S6. TI slopes for MCLs showed a shallower decline of 0.78 dB/doubling the number of pulses, when compared to that of THRs. This was consistent with findings of Zhou et al. (2012) and Obando Leitón (2019), where shallower TI slopes were found for comfortable levels and MCLs, respectively. Nevertheless, given this shallow decline and that TBC is proportional to the power consumption of the implant, our results also show that very high pulse rates (when using biphasic pulses) are not very efficiently stimulating neurons (a schematic illustration of the integration of charges in the 16-pulse condition is depicted in Supplementary Figure S7).

The fact that not only a pulse rate (10,000 pps) but also a burst rate (37 bps) were employed in the study might raise the hypothesis that a combination of both rates, and not only the pulse rate, contributes to temporal integration functions. This needs us to investigate phenomena related to temporal processing of ANFs including refractoriness, facilitation, accommodation, and high-frequency spike rate adaptation (see Boulet et al. (2016) for review). Each of the mentioned phenomena is effective in certain conditions and time ranges. Refractoriness and high-frequency spike rate adaptation are related to conditions where the stimulation amplitude is (well) above thresholds (e.g., MCLs), whereas the facilitation and accommodation deal with subthreshold amplitudes. Refractoriness states that a single nerve fiber has an elevated threshold after firing an action potential (relative refraction period), in a short period after a first action potential it is even impossible to elicit another action potential (absolute refractory period). The duration of the absolute refractory period is around 0.5 ms (Hodgkin and Huxley, 1952; Matsuoka et al., 2001; Boulet et al., 2016); relative refractory period for the auditory nerve is about 4 ms (Boulet et al., 2016). This means that the high pulse rate used in this study (10 kHz) interacts with the refractory time for multi-pulse stimulation. That is, the population of nerves that responded to the first pulse of a multi-pulse burst cannot be activated by further pulses of the burst and instead, only a population other than that responded to the first pulse may respond to the second pulse of the burst.

Spike rate adaptation characterizes the reduced ability of ANs to elicit action potentials in response to pulse trains with relatively high rates (>250 pps). The time course of the spike rate adaptation effect is reported to be between 10 and 100 ms (Zhang et al., 2007; Miller et al., 2011; Boulet et al., 2016), when the stimulation lasts 300 ms, i.e., excitability of neurons starts to decrease immediately after the first spike and then with a time constant between 10 and 90 ms. In this study, although we used a high stimulation rate of 10,000 pps, the stimulation duration was not in the same range of that in abovementioned studies. Therefore, spike rate adaptation has a massive effect on temporal response properties in the present study; it can be concluded that responses are dominated by the first pulse, which is supported by the relatively small changes in MCL amplitudes when the number of pulses was increased. The time course of facilitation and accommodation is reported to be 0.5 ms and between 0.5 and 1 to 10 ms, respectively (Boulet et al., 2016). Therefore, ANFs could integrate residual charge for multi-pulse stimulation, which leads to lower THRs. On the other hand, the inter-burst interval of 27 ms is longer than the 0.5- to 10-ms accommodation window, so that ANF had enough time to recover.

### eABRs to Multi-Pulse Stimulation

**FIGURE 12:**
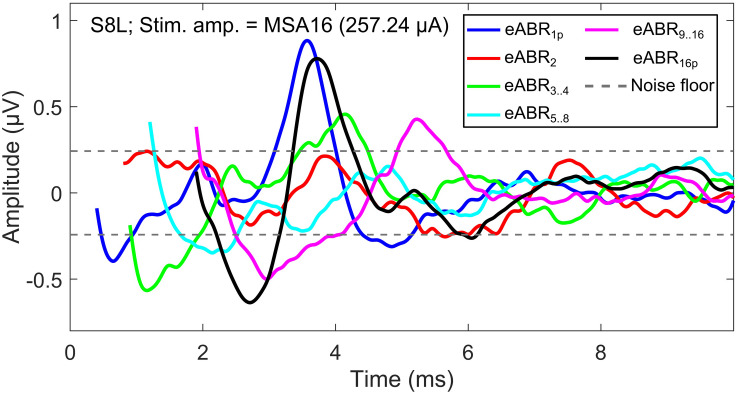
eABRs to individual pulses and groups of pulses in the 16-pulse condition for subject S8L. Note the peaks and troughs of responses to successive pulses and groups of pulses, which suppress the response to the first pulse (eABR_1__*p*_). This destructive interference effect may explain the decrease in the eV amplitude in multi-pulse conditions.

The notion that responses to a high-frequency burst are dominated by the first pulse is also supported by the relatively small changes in eABR responses when the number of pulses increased (Figures 9, 10). The averaged changes in amplitudes were smaller than 2.22 dB and 0.1 ms in latency compared to the single-pulse response with the same amplitude. Figure 9 shows even a decreasing trend for the eV amplitude in MSA4, MSA8, and MSA16 after an initial increase from MSA1 to MSA2, which suggests that the response amplitude falls. Although the stimulation current in each panel of Figure 9 is constant, the number of stimulation pulses, and with it the stimulation TBC, increased. Therefore, higher wave eV amplitudes in response to stronger stimuli would be expected, but this was not observed here. One possible explanation for this observation is destructive interferences, where peaks and troughs of responses to the first pulse are reduced by anti-phasic (because of the delay) responses to later pulses in the train. For instance, the eABR in the 16-pulse condition could be assumed as an arithmetic summation of responses to individual pulses [as in Eq. (3)] or groups of pulses [as in Eq. (4)]. The responses to groups of pulses can be extracted by simple subtractions: for example, the response to the second pulse is *eABR*_2_ = *eABR*_2*p*_−*eABR*_1*p*_ and the response to the third and fourth pulses could be derived as *eABR*_3..4_ = *eABR*_4*p*_−*eABR*_2*p*_, where *eABR*_*ip*_ is the measured eABR to a train of *i*-pulses. Figure 12 depicts such a decomposition of the responses to groups of pulses in the 16-pulse condition for subject S8L. It can be easily observed how the responses to successive pulses, especially *eABR*_*5..8*_ and *eABR*_*9..16*_ (cyan and magenta curves), contribute to suppressing the wave eV amplitude of *eABR*_*1*_ by pushing down the peak of eV of *eABR*_*1p*_ as well as by pulling up its trough, both resulting in a smaller wave eV amplitude of *eABR*_*16p*_. A similar analysis on S8L data in MSA2, MSA4, and MSA8 conditions (not shown) supports the claim that the first pulse of the train has the dominant effect and responses to other pulses suppress the response to the first pulse. Therefore, the drop in eABR wave eV amplitudes of MSA4, MSA8, and MSA16 conditions might not be because of a weaker response but seems likely to be caused by destructive interference with eABR responses to later stimulation pulses. The effect of the destructive interference could be also observed in Figure 11, where the range of eV amplitudes decreased as a function of number of pulses (significant difference only between 2 pulses and 16 pulses) and latencies and their ranges were elevated (significant differences only between 1 pulses and 8 pulses and between 2 pulses and 8 pulses).

(3) $$e\,A\,B\,R_{16\,p}=e\,A\,B\,R_{1}+e\,A\,B\,R_{2}+\ldots +e\,A\,B\,R_{15}+e\,A\,B\,R_{16}$$

(4) $$e\,A\,B\,R_{16\,p}=e\,A\,B\,R_{1}+e\,A\,B\,R_{2}+e\,A\,B\,R_{3..4}+e\,A\,B\,R_{5..8}+e\,A\,B\,R_{9..16}$$

**FIGURE 13:**
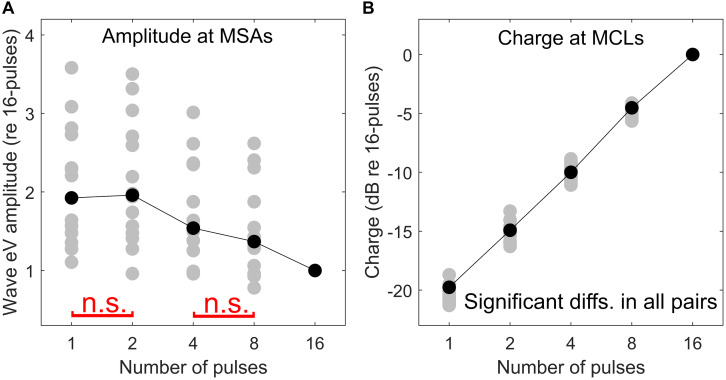
Comparison of psychophysical and eABR results. eABR wave eV amplitude at MSAs (95% of psychophysical MCLs) **(A)** and corresponding stimulation TBCs led to psychophysical MCLs **(B)**. All data are normalized to their corresponding values at the 16-pulse condition. The “n.s.” in red in panel **(A)** shows *not significant* differences between 1 pulse and 2 pulses and between 4 pulses and 8 pulses. The difference between the rest of the pairs was significant. In panel **(B)**, all pairs were significantly different.

Here an additional support for the destructive interference rationale mentioned above is provided. As mentioned in the section “Materials and Methods,” at each multi-pulse condition, eABRs to MSAs, which were defined as 95% of psychophysical MCLs, were measured. Assuming that all MSAs induce the same hearing impression (loudest tolerable level) to each CI subject, similar eABR signals and, consequently, similar wave eV amplitudes are expected. However, as shown in Figure 13A, when the number of pulses increased, the eABR wave eV amplitudes in response to MSAs tended to decrease, but not to preserve. The opposite trends in stimulation TBCs (Figure 13B) and wave eV amplitudes (Figure 13A) also support the rationale of destructive interference, as more TBC would mean more activated ANFs and, consequently, larger eV amplitudes. Additionally, such a destructive effect was found to reverse the tendency of latency, where normally shorter latencies are expected for higher stimulation amplitudes. Figure 10, however, suggests longer-wave eV latencies (maximum of about 0.1 ms) over all subjects, when the number of pulses increased.

### Efficacy of Multi-Pulse Stimulation

For electric biphasic stimulation, pulse shape could affect the detection THRs at the level of a single ANF, eCAPs, or eABRs. It is known that pulses with longer phase durations evoke stronger neural responses when compared to pulses with shorter durations and equal-stimulation amplitude. This means that, in comparison to shorter phases, pulses with longer phases need less current to reach THR. However, according to the fact that the nerve membrane functions more as a leaky integrator rather than a perfect one, pulses with longer phases seem to be less efficient than those with shorter phase durations of the same overall charges (Abbas and Brown, 1991; Shepherd et al., 2001). For single pulses, Moon et al. (1993) observed mean slopes of −3.60 and −5.71 dB/doubling of phase duration when pulse duration was less or more than 0.5 ms/phase, respectively. The effect of phase duration on eCAP and eABR was also found to be correlated with auditory nerve survival in guinea pigs (Prado-Guitierrez et al., 2006). Shepherd and Javel (1999) investigated the efficacy of pulses of different shapes. They found that not only ordinary biphasic pulses but also chopped pulses could make a single ANF elicit an action potential. Shepherd and Javel (1999) also found that charge packages of 2 × 30, 3 × 20, and 6 × 10 μs of same polarity, followed by a series of reversed polarity, could charge the nerve membrane even up to eliciting an action potential. This packet structure, which was called a “chopped pulse,” was found to show 1.5-dB higher THRs (less efficient) than a 60-μs/phase biphasic pulse with a 60-μs interphase gap and, interestingly, at least about 1.5 dB lower THRs (more efficient) when compared to a 60-μs/phase biphasic pulse without interphase gap.

Although the electric current and charge are closely related, in electric hearing, the current, rather than the charge, plays the main role in stimulating auditory nerves. Moreover, in MED-EL implants there is a coupling capacity, which forces the net charge to be zero. A net residual potential of the electrodes should have no effect in the resistive fluid. In such a structure, if the stimulation mode was 100% efficient, it could be expected that the total charge required to elicit THR/MCL remained constant. In such a condition, the stimulation amplitude in an *m-pulse* condition should decrease by a factor of $\frac{1}{m}$, compared to the 1-pulse condition. This was not found in the data of the present study. Figure 6 highlights the inefficiency of multi-pulse stimulation. The TBC of the positive phases in a multi-pulse condition is plotted as a function of the number of pulses for THR and MCL. In both THR and MCL data (Figure 6B), the TBC needed to elicit THR/MCL increased drastically as a function of the number of pulses (see also Supplementary Figure S7). The steeper slope for THRs shows a stronger inefficiency compared to that for MCLs. The inefficiency found in this study can be attributed to rapid phase switching of pulses; therefore, multi-pulse stimuli are far less efficient than single pulses.

### Temporal Effects in eABRs to Fast Pulse Trains

Since all multi-pulse stimuli used in the eABR section of this study were (well) above THR, temporal phenomena such as facilitation and accommodation would not be involved in temporal processing of ANFs. Refractoriness and depression, however, are likely occurring phenomena and the eABR measurements might shed light on these effects. Abbas and Brown (1991) employed a masker-probe paradigm in which an initial pulse, termed masker, followed by a second pulse, named probe, with varying inter-pulse intervals from the masker was used to measure eABRs. They found that average durations of 5.10 and 4.63 ms, respectively, were needed for the probe (second) pulse to fully recover, using two different CI types. Their findings seem to be consistent with the relative refractory period of about 4 ms, as reported in Boulet et al. (2016). This also suggests that, in the 16-pulse condition of the present study, where the stimulation lasted for 1.6 ms, a portion of the ANFs might fire twice during the train. This portion would probably be those ANFs which responded to the first pulses and, later, most likely to the pulses close to the end of the train, due to their recovery after their absolute refractory period.

Particularly in multi-pulse stimulation employed in this study, the initial pulse activated a population of ANFs, which consequently led to a detectable eABR in the brainstem. This population is not capable of responding to the second pulse and has only limited responses during the rest of the pulses in the burst, because of the refractoriness. Therefore, another population of ANFs, other than the one that responded to the first pulse and presumably farther than that, might be capable of eliciting action potentials as a response to the second pulse. In case the second pulse alone is not strong enough, a group of pulses might be able to make ANFs fire, as described in Eq. (4). Generalized to further pulses, characteristics of wave eV amplitudes in response to multi-pulse stimulation provide insight into how multi-pulse stimuli are integrated at the level of the brainstem and they might be a potential measure of health state and/or survival of ANFs.

Bai et al. (2019) and Obando Leitón (2019) confirmed that stimulation of a single electrode of the CI leads to a broad spread of current along the cochlea, which means the auditory nerves are stimulated not only by the nearest electrode but also by a number of neighboring electrodes. This would mean that in the CIS strategy the effective stimulation rate in electric hearing is not the rate of individual electrodes but a burst with the global stimulation rate originating from neighboring electrodes with overlapping current spread. Considering a typical stimulation rate of 800–2000 pps for individual electrodes, the high stimulation rate of 10,000 pps used in this study represents the global stimulation rate induced by stimulation of N neighboring electrodes. Thus, eABRs in response to multi-pulse stimuli of high rate could be used for estimation of THRs like those used in clinics. This assumption of course requires further investigation.

## Data Availability Statement

All datasets generated for this study are included in the article/Supplementary Material.

## Ethics Statement

The studies involving human participants were reviewed and approved by the Klinikum rechts der Isar der Technischen Universität München. The patients/participants provided their written informed consent to participate in this study.

## Author Contributions

AS contributed to the study design, data collection, data analysis, and manuscript drafting. WH contributed to the study design and critical manuscript revision. All authors contributed to the article and approved the submitted version.

## Conflict of Interest

The authors declare that the research was conducted in the absence of any commercial or financial relationships that could be construed as a potential conflict of interest.
