# Supplementary figures and images for: Investigation of Electrically Evoked Auditory Brainstem Responses to Multi-Pulse Stimulation of High Frequency in Cochlear Implant Users
Source: Front Neurosci. 2020 Jun 30;14:615. doi: 10.3389/fnins.2020.00615 (PMC7338891; doi:10.3389/fnins.2020.00615)

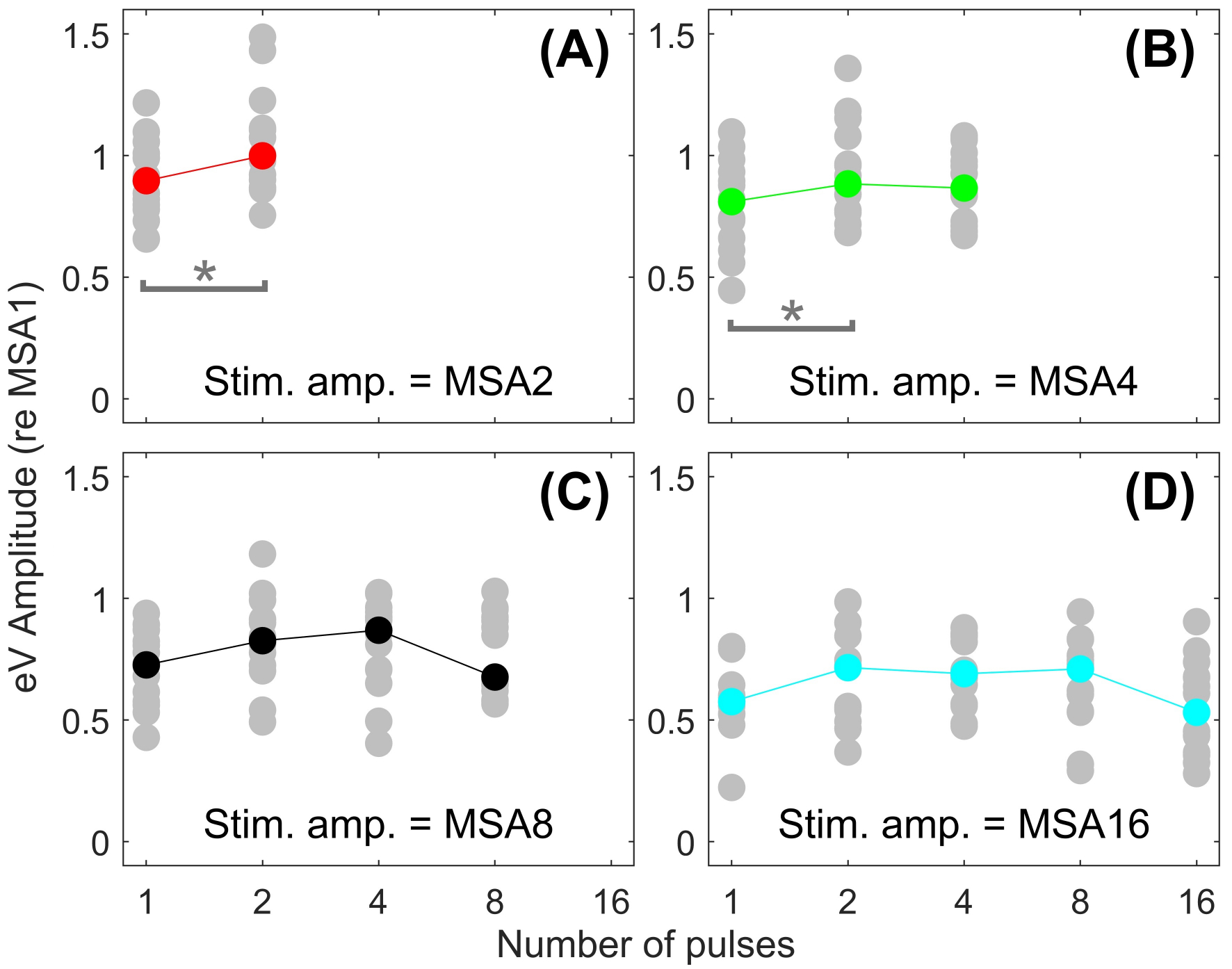

Supplement: Supplementary file 1 [file Data_Sheet_1.ZIP › Supp_Material_Saeedi/Fig_9_Amplitude_all_subjects_all_MP_conditions_re_MSA1_star.jpg]

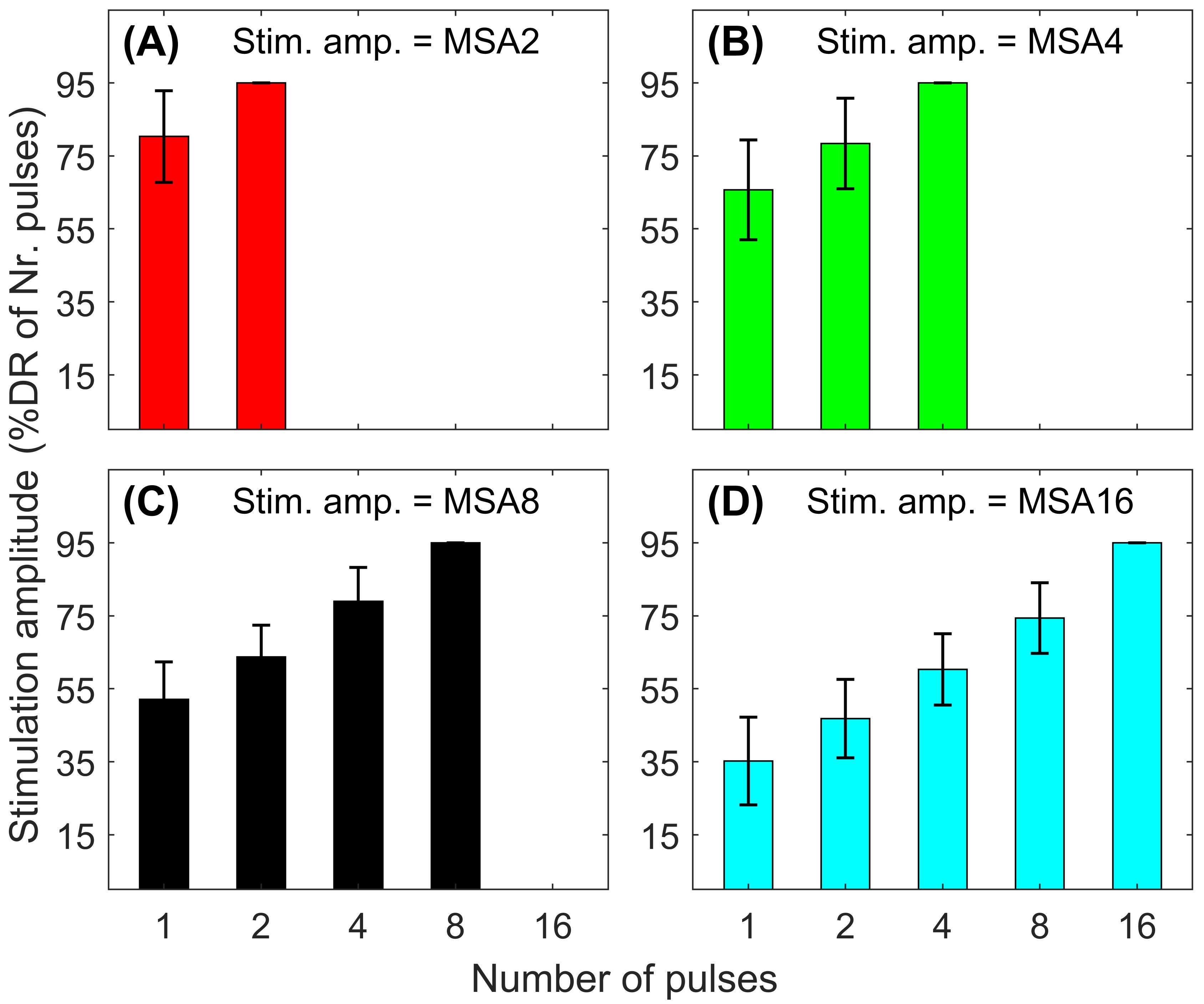

Supplement: Supplementary file 1 [file Data_Sheet_1.ZIP › Supp_Material_Saeedi/SuppMat_Fig_1_All_relative_MSAs.jpg]

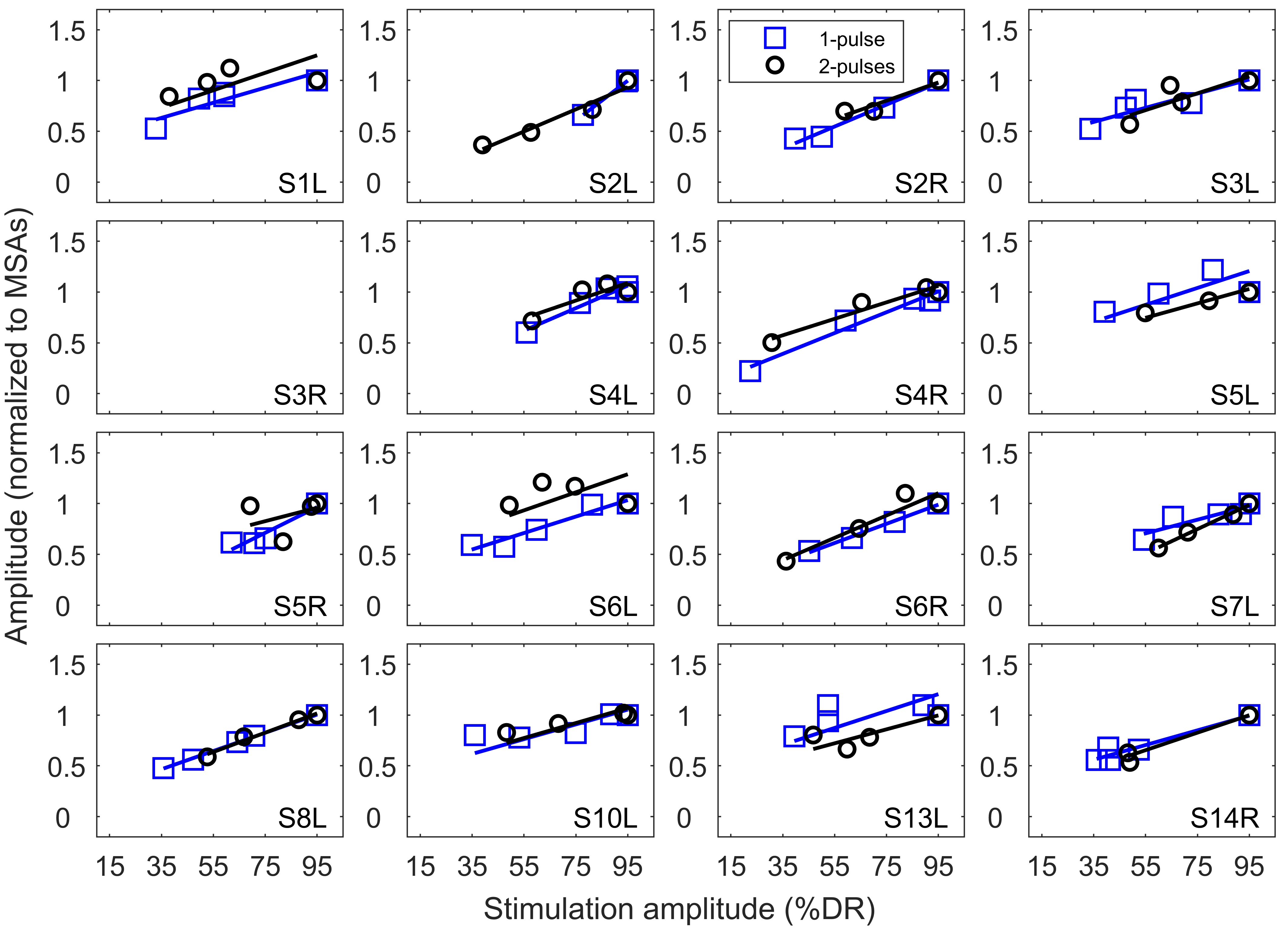

Supplement: Supplementary file 1 [file Data_Sheet_1.ZIP › Supp_Material_Saeedi/SuppMat_Fig_2_All_slopes_1pulse_2pulses.jpg]

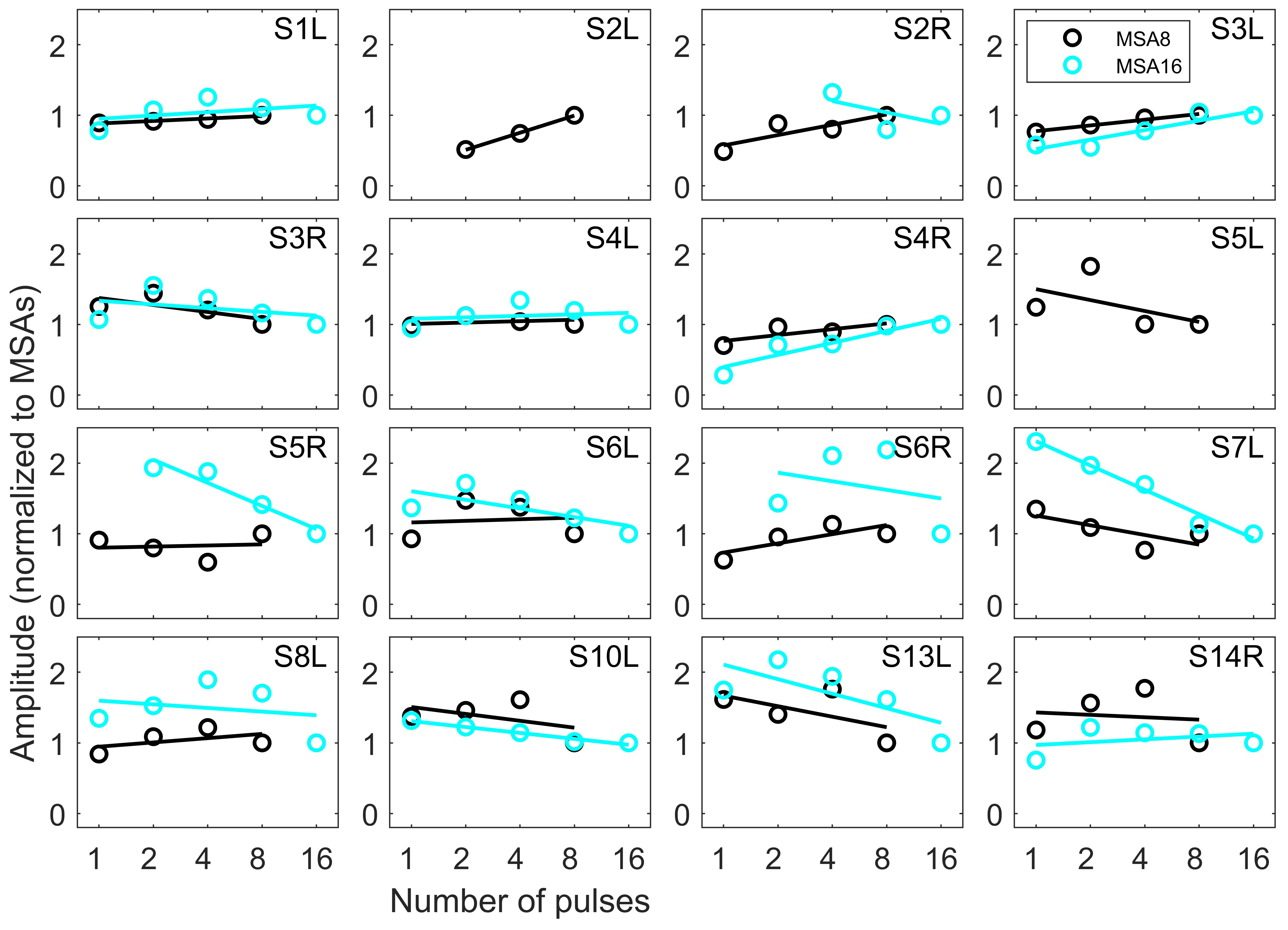

Supplement: Supplementary file 1 [file Data_Sheet_1.ZIP › Supp_Material_Saeedi/SuppMat_Fig_3_All_slopes_MSA8_MSA16.jpg]

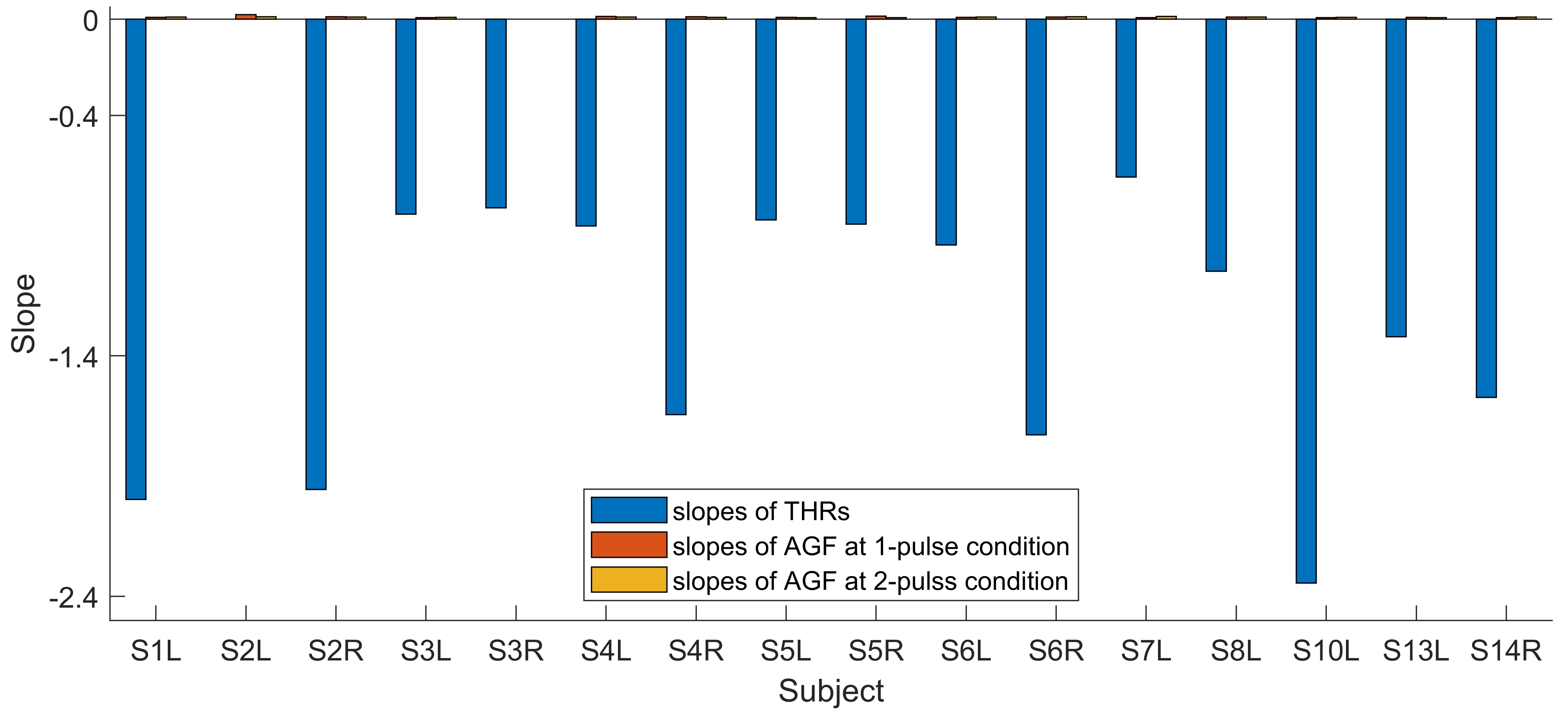

Supplement: Supplementary file 1 [file Data_Sheet_1.ZIP › Supp_Material_Saeedi/SuppMat_Fig_4_All_slopes_THR_1p_2p_comparison.jpg]

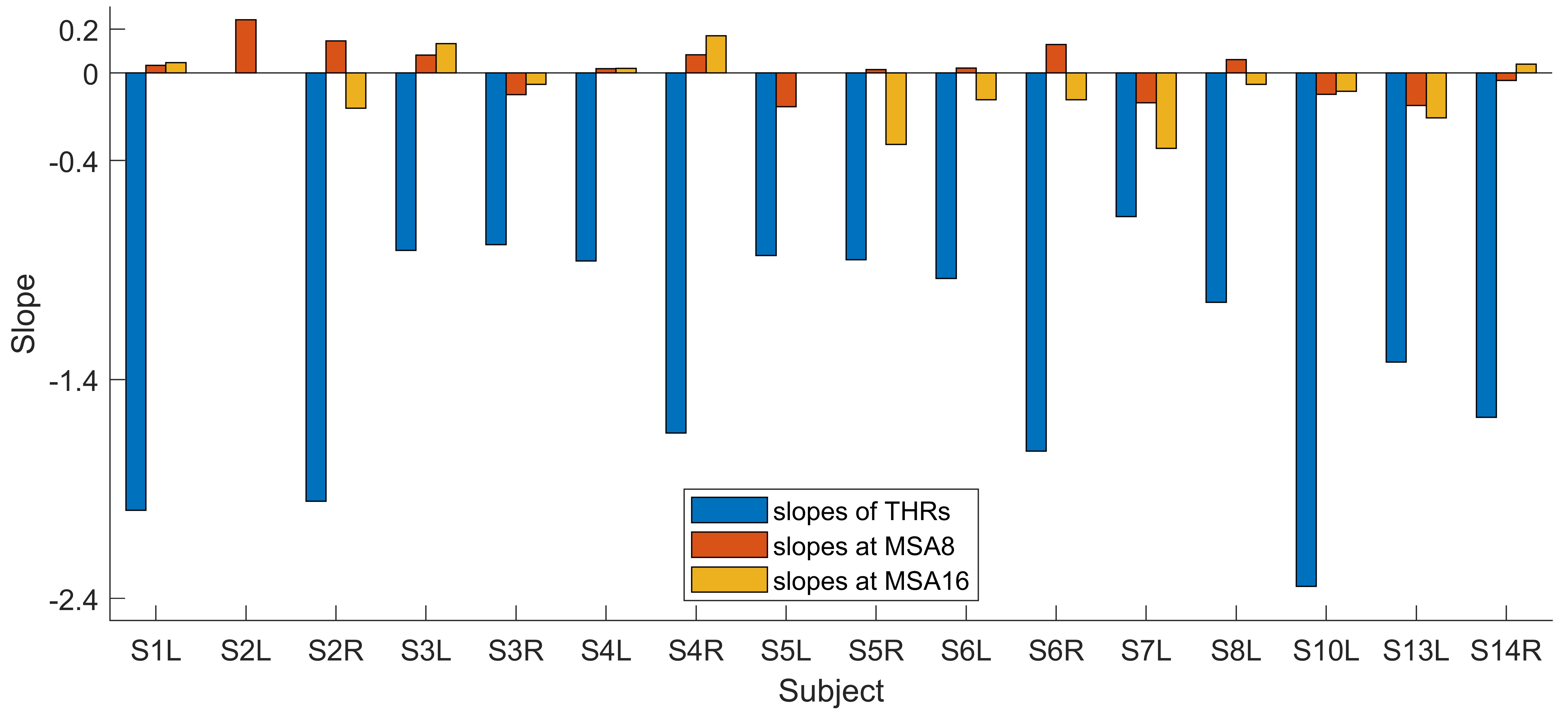

Supplement: Supplementary file 1 [file Data_Sheet_1.ZIP › Supp_Material_Saeedi/SuppMat_Fig_5_All_slopes_THR_MSA8_MSA16_comparison.jpg]

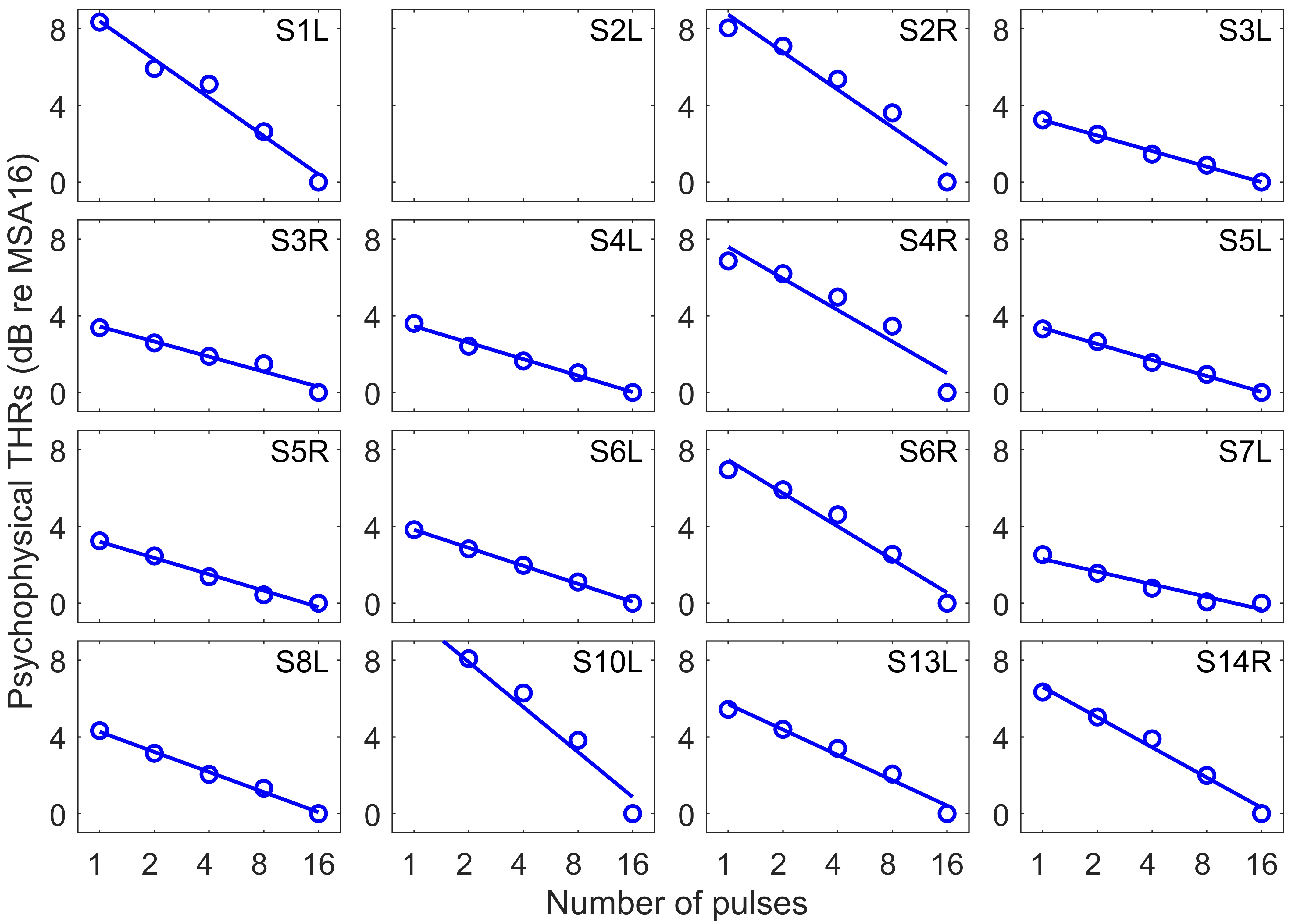

Supplement: Supplementary file 1 [file Data_Sheet_1.ZIP › Supp_Material_Saeedi/SuppMat_Fig_6_All_slopes_THRs_re_MSA16.jpg]

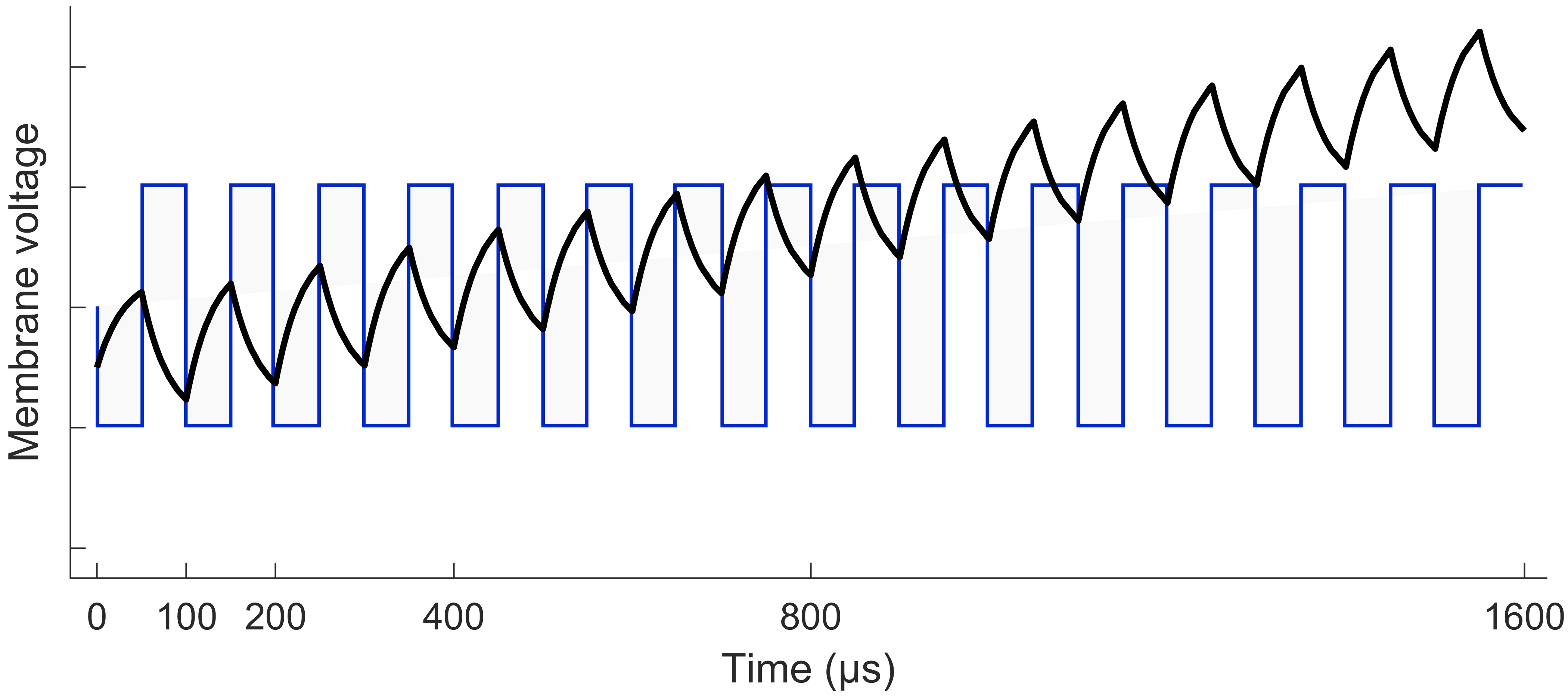

Supplement: Supplementary file 1 [file Data_Sheet_1.ZIP › Supp_Material_Saeedi/SuppMat_Fig_7_schematic_multi-pulse_integration.jpg]
